# Metal Levels in Striped Dolphins (*Stenella coeruleoalba*) and Common Dolphins (*Delphinus delphis*) Stranded along the Sicilian Coastlines of the Mediterranean Sea

**DOI:** 10.3390/ani14142063

**Published:** 2024-07-14

**Authors:** Clara Naccari, Vincenzo Ferrantelli, Gaetano Cammilleri, Francesco Giuseppe Galluzzo, Andrea Macaluso, Pietro Riolo, Gianluigi Maria Lo Dico, Roberto Bava, Ernesto Palma

**Affiliations:** 1 Department of Health Sciences, University “Magna Græcia” of Catanzaro, 88100 Catanzaro, Italy; roberto.bava@unicz.it (R.B.); palma@unicz.it (E.P.); 2 Istituto Zooprofilattico Sperimentale della Sicilia “A. Mirri”, 90129 Palermo, Italy; vincenzo.ferrantelli@izssicilia.it (V.F.); gaetano.cammilleri86@gmail.com (G.C.); francesco.galluzzo@izssicilia.it (F.G.G.); andrea.macaluso@izssicilia.it (A.M.); pietro.riolo@izssicilia.it (P.R.); gianluigi.lodico@izssicilia.it (G.M.L.D.); 3 Interdepartmental Service Center—Center for Pharmacological Research, Food Safety, High Tech and Health (CIS-IRC-FSH) University “Magna Græcia” of Catanzaro, 88100 Catanzaro, Italy

**Keywords:** toxic and essential metals, organs/tissues, *Stenella coeruleoalba*, *Delphinus delphis*, Mediterranean Sea

## Abstract

**Simple Summary:**

Dolphins, top predators able to accumulate high levels of environmental contaminants, are used as sentinel species of marine pollution. In this study, the content of metals and metalloids (Hg, Pb, Cd, As, Se and Zn) has been evaluated through ICP-MS analysis in several organs/tissues (liver, muscle, lung, kidney and skin) of striped dolphins (*Stenella coeruleoalba*) and common dolphins (*Delphinus delphis*) stranded along the Sicilian coastlines of the Mediterranean Sea. The results confirm the exposure of both dolphin species to metals and metalloids, with the highest Hg content correlated to the pollution of the environment where they lived. Significant differences were observed in metals distribution in different organs/tissues analyzed and confirmed by the comparative analysis of metals according to sex and state of development of both dolphin species. These data underline the important role of *Stenella coeruleoalba* and *Delphinus delphis* as sentinels of the aquatic environment for assessment of the trend of metals pollution in coastal ecosystems and, indirectly, the health of aquatic species of the Mediterranean Sea.

**Abstract:**

Dolphins, top predators of the aquatic food chain, are used as sentinel species of marine pollution as they are sensitive to environmental changes and able to accumulate a large content of contaminants. Several EU directives promote study of marine mammalians as bio-indicators to evaluate the presence of contaminants in the aquatic environment, such as the Mediterranean Sea, which is rich in environmental pollutants due to its geographic and geo-morphological characteristics. The aim of this study was to evaluate the content of toxic and essential metals and metalloids (Hg, Pb, Cd, As, Se and Zn), through ICP-MS analysis, in organs/tissues (liver, muscle, lung, kidney and skin) of striped dolphins (*Stenella coeruleoalba*) and common dolphins *(Delphinus delphis*) stranded along the Sicilian coastlines of the Mediterranean Sea. The results confirm the exposure of dolphins to toxic metals and metalloids, with the highest Hg levels observed in skin and liver, although a low Metal Pollution Index (MPI) was found in all samples of both dolphin species. From a comparative analysis of trace metals and metalloids according to sex and state of development, the highest levels of Cd and As were found in females vs. males and adults vs. juveniles, except for Pb in both species, and significant differences were observed between the two species, size of specimens, and organs/tissues analyzed. The highest Hg levels were correlated to those of essential metals Se and Zn, expressed as molar ratios, to evaluate the potential synergic effect of these detoxifying elements against Hg toxicity. This study confirms the rule of *Stenella coeruleoalba* and *Delphinus delphis* as valid sentinel species of the Mediterranean Sea, to verify the trend of metals pollution in this aquatic environment and, consequently, the health of these marine species.

## 1. Introduction

Dolphins, top predators of the aquatic food chain, can be considered sentinel species of marine pollution because they are long-lived animals, sensitive to environmental changes, long-term coastal residents, and can easily accumulate contaminants (heavy metals, PCBs, OCs, dioxins, POPs, etc.) due to their mobility in sea water over long distances [1,2,3,4]. These marine sentinels are very useful for evaluation of the health of the aquatic ecosystem and, at the same time, the impact of pollutants on animals living in coastal areas. In addition, dolphins can be considered indirectly useful sentinels for public health and risk assessment for consumers due to their food habits, as they feed on fishery products commonly consumed by Mediterranean populations [5,6].

The striped dolphin (*Stenella coeruleoalba*) is the most widespread cetacean species in the waters of the Mediterranean Sea. This marine environment represents its ideal habitat: *S. coeruleoalba* moves near the coast to hunt during the night and prefers the open sea during the day to rest. It can live in both temperate and tropical waters, dive to a depth of 200 m for hunting, and could be exposed to high levels of pollutants according to the trophic transfer factor of the food chain, feeding on cephalopods and meso-pelagic fishes and crustaceans [6,7], which are good accumulators of heavy metals present in sediment, the sea bottom, and/or superficial waters [7]. In recent years, this species has been subject to a significant population decline due to several threats, such as marine environmental pollution, climatic changes, fishing activities and infectious diseases, particularly the *Morbillivirus* epidemic in the 1990s. It was hypothesized that exposure to high levels of pollutants such as heavy metals, PCBs, DDT, oil and gas extracts, etc., in the marine environment where they live could be responsible for immune-suppression of *Stenella* spp., making it susceptible to infections such as *Morbillivirus* and *Toxoplasma* [8,9].

The common dolphin (*Delphinus delphis*) is most abundant in the Atlantic Ocean, whereas in the Mediterranean area it is present mainly in the Tyrrhenian Sea. This species prefers temperate waters and can be found both inshore and offshore; it lives in large groups of 30–50 individuals and sometimes also with striped dolphins, particularly when travelling and feeding. It mainly feeds on cephalopods and small fishes present in its habitat, such as sardines and anchovies [10]. For *D. delphis* there are also important survival risks due to interactions with the fishing industry and environmental pollution of their marine habitat, where they are easily exposed to various contaminants. 

The Mediterranean Sea, due to its geographic and geo-morphological characteristics, is particularly rich in environmental pollutants (naturally occurring or due to anthropic activities) and due to the limited movement of waters they can accumulate in this basin, contributing to chronic exposure in marine species [11]. Among marine contaminants, great attention is focused on heavy metals [12], elements naturally occurring in the environment through terrestrial crust erosion, volcanic activity, dust deposition, etc., and also released as a consequence of anthropic activities (fossil fuels combustion, exhaust gases, agricultural practices, industrial activities, incineration of solid wastes, etc.). These pollutants can reach marine waters through atmospheric precipitation and waste-water discharges and, consequently, enter the aquatic food chain, accumulating in small fishes, predators and marine mammalians, with a negative impact on the marine ecosystem and the health of aquatic species [1,2,3,4,13,14,15].

Several studies have reported toxic effects of these contaminants on dolphins, such as immune-suppression, renal and hepatic damage, metal-induced mutagenic alterations, and interference in neurological processes [16]. It has also been hypothesized that dolphins may play a role as a potential model for study of human neuropathies induced by environmental contaminants, such as heavy metals and methyl-mercury [17,18], which are able to bioaccumulate and, consequently, biomagnify in dolphin tissues. Finally, these marine mammalians could also be considered useful in risk assessment for consumers of fishery products [6].

Dolphins are reported in the Convention for the Protection of the Mediterranean Sea Against Pollution and in the United Nations Environment Program Mediterranean Action Plan (21 May 2021) because there have been numerous sightings of beached dolphins on different coastlines around the world due to various causes of death (viral infections and parasitic diseases, tumors, toxic substances, plastic bags that obstruct the intestine, injuries due to interaction with fishing activities, etc.). At the same time, there are several EU Directives on protection of mega-fauna and the marine environment, such as the Marine Strategy Framework Directive (MSFD) [19], which promotes monitoring studies of marine mammalians as bioindicators, to evaluate the presence of contaminants in different aquatic environments and the health of these marine species.

In our previous investigation [4], the complete mineral profile in *S. coeruleoalba* was documented. This species is used as a sentinel species to evaluate not only marine environmental pollution but also dolphin health status.

In this study, the content of toxic and essential metals and metalloids was evaluated in different organs/tissues of two dolphin species, *S. coeruleoalba* and *D. delphis*, stranded along the Sicilian coastlines of the Mediterranean Sea. In particular, a comparative analysis of the metals and metalloids levels was carried out according to sex and state of development of dolphins and a statistical analysis was conducted considering the two species, size, and organs/tissues analyzed, to evaluate possible differences in metals exposure. In addition, considering the role of dolphins as sentinel species, we analyzed the Metal Pollution Index (MPI) to evaluate marine environmental pollution, and the molar ratios Se/Hg and Zn/Hg for risk assessment of dolphin health. 

## 2. Materials and Methods

### 2.1. Reagents

All solvents used, ultrapure water (resistivity of 18 MΩ cm), HNO_3_ (70% *v*/*v*) and H_2_O_2_ (30% *v*/*v*) for trace metal analysis, were provided from J.T. Backer (Mallinckrodt Backer, Milan, Italy); stock standard solutions (1000 mg L^−1^ in 2% nitric acid) of each mineral element and stock solutions of on-line internal standards Sc, Ge, In, Bi (1000 mg L^−1^ in 2% nitric acid) from Fluka (Milan, Italy).

### 2.2. Sample Collection

The present investigation was conducted on 25 dolphins (13 *Stenella coeruleoalba* and 12 *Delphinus delphis*) stranded along the Sicilian coasts of the Mediterranean Sea, collected after death by the Istituto Zooprofilattico Sperimentale della Sicilia “A. Mirri”, Palermo (Italy). From these dolphins, different organs were taken according to the state of conservation, and particularly from *S. coeruleoalba* liver, muscle, lung and skin were taken, while from *D. delphis* liver, muscle, kidney and skin samples were taken. The specimens were of different sex, weight, length and state of carcass conservation, as reported in Table 1. Considering that no specific data are available relating to age, length was taken as a replacement for age [20,21]: length between 0.95–1.80 m was considered indicative of juveniles while >1.81 m was considered indicative of adults (Table 1). Among all dolphins, three samples showed back injuries, another two exhibited severed tail, probably indicative of death by interaction with fishing activities; as for the others, it was difficult to identify the causes of death due to absence of particular signs on the carcass and evidence from microbiological analysis and necropsies. All samples were collected taking care to avoid contamination and, prior to analysis, were stored at −20 °C in PET containers.

### 2.3. Sample Mineralization

From each dolphin, all organs and tissues were collected and homogenized; then, aliquot of 0.5 g wet weight (w.w.) from each one was mineralized with HNO_3_ (70%) and H_2_O_2_ (30%) in a closed-vessel microwave digestion system (CEM Microwave^TM^ Digestion System, Discovery SP-D, CEM Corporation, Matthews, NC, USA), and finally submitted to ICP-MS analysis for metals determination [7]. Analytical blanks were prepared in a similar manner and all determinations were performed in triplicate. Before the analysis, the glass-ware was washed with HNO_3_ (15%) overnight, rinsed with ultrapure water and stored dry to prevent contamination.

### 2.4. ICP-MS Analysis

For the determination of metals and metalloids (Hg, Pb, Cd, As, Se and Zn) the instrument used was an ICP-MS spectrometer equipped with an auto-sampler ASX520 (Cetac Technologies Inc., Omaha, NE, USA), operating according to the following conditions: RF power, 1550 W; plasma gas flow rate, 14 L min^−1^; auxiliary gas flow rate, 0.89 L min^−1^; carrier gas flow rate, 0.91 L min^−1^; helium collision gas flow rate, 4.5 mL min^−1;^ spray chamber temperature, 2.70 °C; sample depth, 4.27 mm; sample introduction flow, 0.93 mL min^−1^; nebulizer pump, 0.1 rps; extract lens 1 voltage, 1.5 V [4]. For acquisition of data, the instrument was used in He KED mode, removing spectral interference due to low and high mass elements. Monitored isotopes were ^202^Hg, ^208^Pb, ^111^Cd, ^75^As, ^82^Se and ^66^Zn, while for on-line internal standards ^45^Sc, ^72^Ge, ^209^Bi, ^115^In were selected.

### 2.5. Validation of Analytical Method

As reported in Table 2, the accuracy and repeatability of the method were assessed through the analysis of certified reference materials DOLT-5 (Dogfish Liver Reference Material for Trace Metals, National Research Council of Canada). To assess the precision of the method, the relative standard deviation (%) of four independent determinations was used; blank controls were analyzed together with each batch of samples and used to exclude interference and/or contamination during the analysis (see Table 2).

### 2.6. Parameters to Assess Metals Pollution

From the analysis of the trace elements content in both species, the Metals Pollution Index (MPI) was calculated, a parameter used for the assessment of marine metal pollution, which expresses the metals accumulation levels in organs and tissues [22], using the following formula:

*MPI* = (*M*1 × *M*2 × *M*3 ×…*Mn*) ^1/*n*^ (1)

where *Mn* represents the concentration of “*n*” metal (µg g^−1^) found in a tissue sample.

The molar ratios of ^82^Se/^201^Hg and ^66^Zn/^201^Hg were calculated as the ratio of atomic masses [12], to correlate the content of toxic and essential metals, and a value of 1 was referred to a protection index.

### 2.7. Statistical Analysis

All data obtained in this study were reported as mean values ± S.D., calculated over three determinations. To study the normality of the data set a Shapiro-Wilk test was used. A Wilcoxon rank sum test was used to verify metals and metalloids differences between the two species analyzed. Furthermore, Spearman’s rank correlation test was applied to calculate the correlation between trace metal(loid)s contents in all organs and tissues analyzed and the morphometric parameters (weight and total length); however, samples of *D. delphis* were excluded from the correlation analysis due to the high presence of tied values. The differences were considered statistically significant for *p <* 0.05. In the case of values below detection limit, these were set to half of LOD values [23,24]. All the statistical analyses were made with R 4.2.2 freeware (https://cran.r-project.org/ accessed on 20 March 2024); the ggplot2 version 3.4.2, the ggpubr 0.6.0.99, and the ggstatplot packages were employed for data visualization [25].

## 3. Results

The toxic and essential metals and metalloids content found in various organs/tissues (liver, muscle, lung, kidney and skin) of the two dolphin species analyzed are reported in Table 3. Among toxic metals and metalloids, Hg was the most abundant in all organs/tissues, followed by As, Pb, and Cd lowest, with higher values in *S. coeruleoalba* than *D. delphis* (*p <* 0.01 and 0.05, respectively). The essential metals Se and Zn showed a similar trend in both dolphin species.

From a specific statistical analysis on the normality of data groups with the Shapiro-Wilk test, significant differences were observed in muscle for As (*p <* 0.001) and Cd (*p <* 0.001) of *D. delphis* vs. *S. coeruleoalba*.

**Figure 1:**
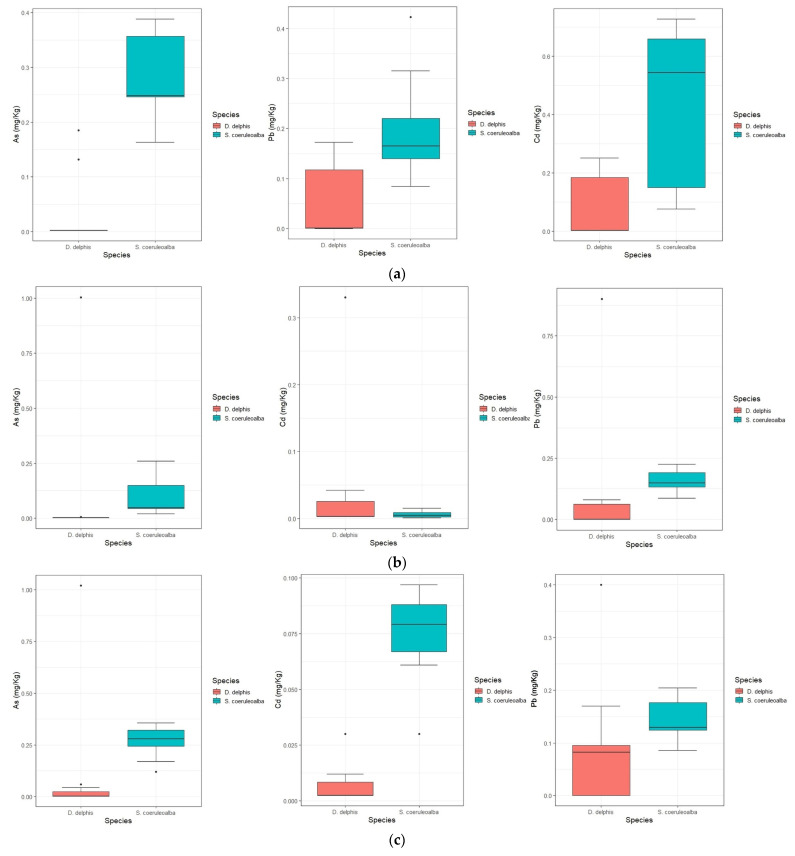
Box whisker plots of the metals and metalloids distribution in liver (**a**), muscle (**b**) and skin (**c**) of *Stenella coeruleoalba* and *Delphinus delphis*. Circles correspond to outliers.

Considering, instead, the comparison relating to the distribution of metals and metalloids content in the two dolphin species with the Wilcoxon rank test, statistically significant differences were observed in muscle for As (*p <* 0.001) and Pb (*p <* 0.001); in liver for As (*p <* 0.001), Cd (*p <* 0.001) and Pb (*p <* 0.001); and in skin for As (*p <* 0.001), Cd (*p <* 0.001) and Pb (*p <* 0.001) (Figure 1).

**Figure 2:**
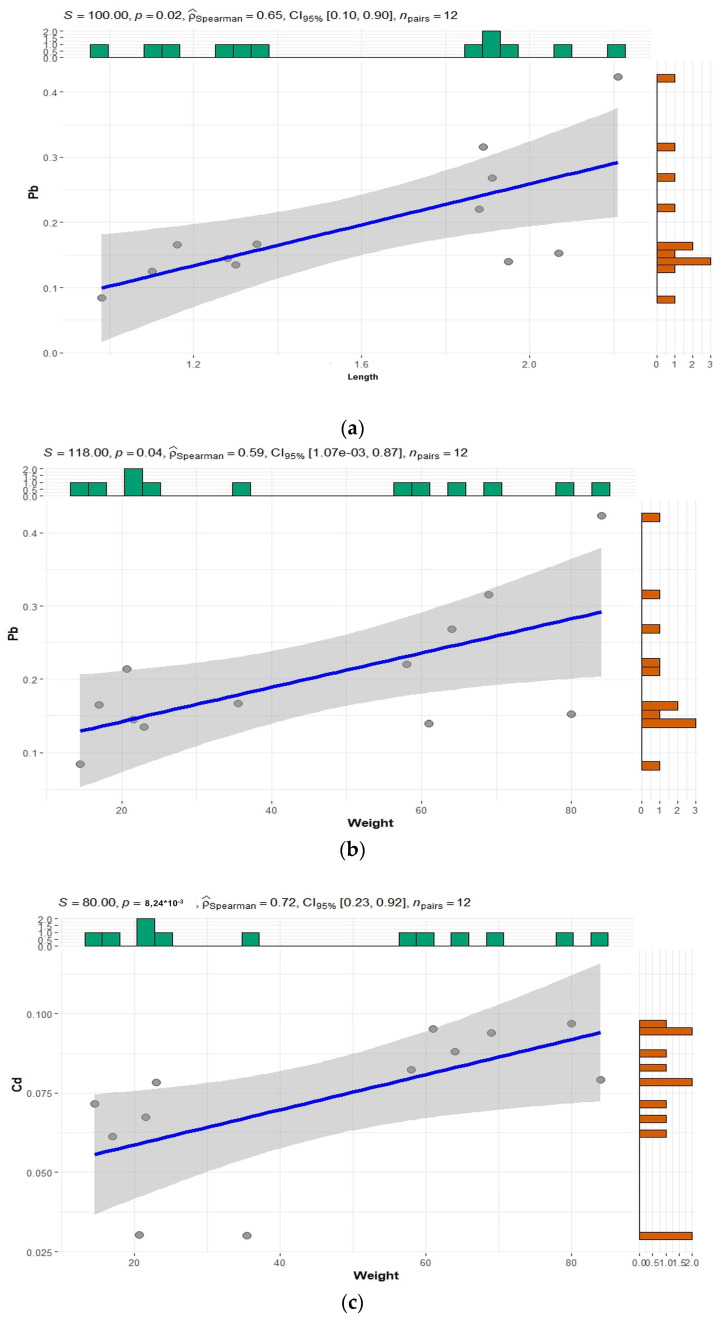
Spearman tests correlation between Pb content in liver and length (**a**) and weight (**b**) and between Cd levels in skin and weight (**c**) of *S. coeruleoalba*.

Relating to the correlation among metals and metalloids levels and morphometric parameters (length and weight), no significant correlation was observed for *D. delphis* samples, due to the high presence of tied values; differences, instead, were found in *S. coeruleoalba*. Particularly, the Spearman tests showed a positive significant correlation between Pb content in liver and the length and weight of the *S. coeruleoalba* samples analyzed, and also between Cd in skin and weight (Figure 2).

**Figure 3:**
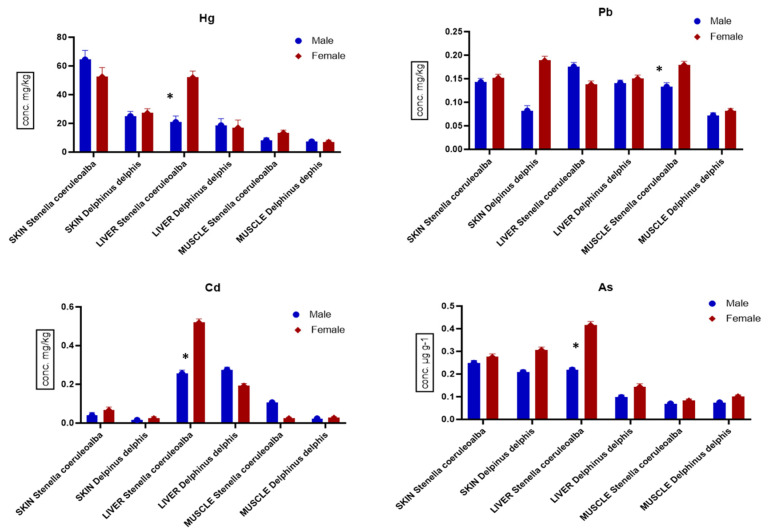
Comparison among levels of Hg, Pb, Cd and As (μg g^−1^ w.w. ± S.D.) in skin, liver and muscle of male and female samples of *S. coeruleoalba* (male n = 6; female n = 7) and *D. delphis* (male n = 5; female n = 7). Significance was accepted for * *p <* 0.05.

The comparative analysis of toxic metals and metalloids found in skin, liver and muscle of males and females of *S. coeruleoalba* and *D. delphis* is reported in Figure 3. In both species, the highest concentrations of metals were present in organs of female samples. Particularly, highest levels of As and Pb in skin were found in female vs. male samples, with statistically significant differences in *D. delphis* (*p <* 0.05). In liver, the highest levels of Cd and As were observed in females vs. males (*p <* 0.01 and *p <* 0.05, respectively) of *S. coeruleoalba*. In muscle, the highest levels of Pb were observed in females vs. males of *S. coeruleoalba* (*p <* 0.05) while As was similar in both dolphin species.

**Figure 4:**
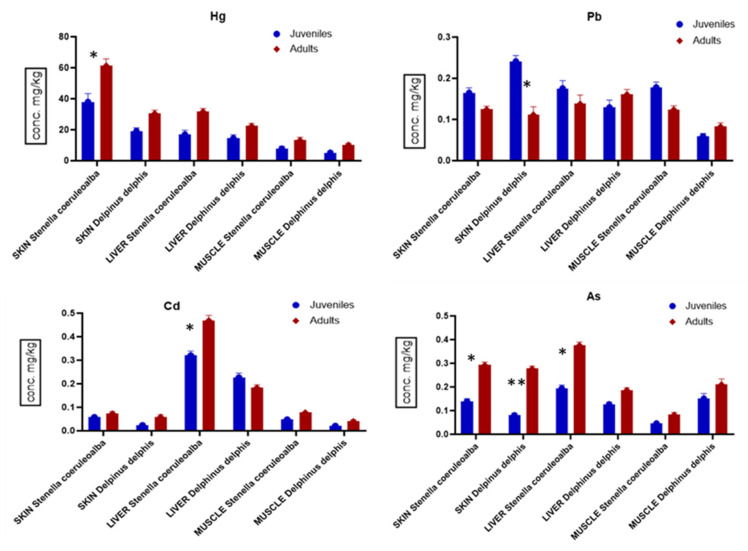
Comparison among levels of Hg, Pb, Cd and As (μg g^−1^ w.w. ± S.D.) in skin, liver and muscle of juveniles and adults of *S. coeruleoalba* (juveniles n = 6; adults n = 7) and *D. delphis* (juveniles n = 5; adults n = 7). Significance was accepted for * *p <* 0.05 and ** *p <* 0.001.

Similarly, a comparative analysis of toxic metal and metalloids in skin, liver and muscle was carried out relating to the developmental stage (juveniles vs. adults) of striped and common dolphins (Figure 4). In detail, the concentrations in skin showed a similar trend in both species, with the highest level of Pb in juveniles (*p <* 0.05) and As in adults (*p <* 0.01). In liver, the highest levels of metals were found in *S. coeruleoalba*, particularly Cd and As in adult vs. juvenile samples (*p <* 0.01). In muscle, Pb levels were highest in juveniles vs. adults of *S. coeruleoalba* (*p <* 0.05), while As was highest in adults vs. juveniles of *D. delphis* (*p <* 0.05). 

Considering that the highest levels of Hg were present in all organs/tissues compared to other elements (Table 3), for an evaluation of the toxicological risk, they were correlated with those of Se and Zn, which are essential elements with detoxification activity, referred to equimolar ratio 1 (protection index) [12]. The molar ratios of ^82^Se/^201^Hg and ^66^Zn/^201^Hg were reported in Table 4, where it is possible to observe that values were lower than 1 in all organs/tissues, except for kidney of *D. delphis*. In detail, the lowest molar ratios were found in skin and liver of both species; however, in muscle of both species the ratio ^66^Zn/^201^Hg was near to 1.

**Figure 5:**
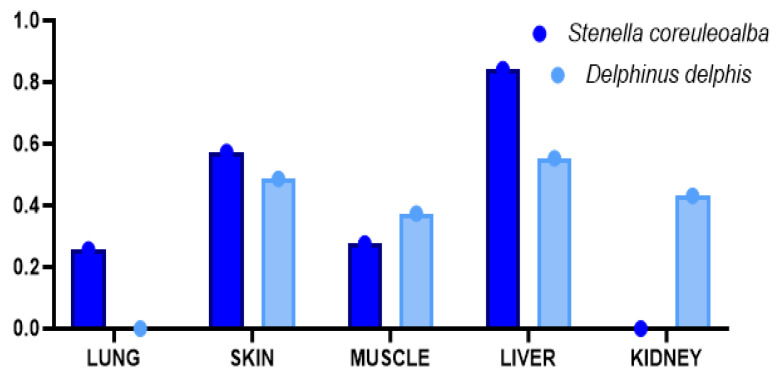
Metal Pollution Index in samples of *S. coeruleoalba* and *D. delphis* from the Mediterranean Sea.

Considering all metals and metalloids found in various organs/tissues, the Metal Pollution Index was also evaluated (Figure 5) in both dolphin species; despite the high Hg levels, the values of MPI were <1 (safety levels) [26,27,28].

Finally, the results of this study were compared with those of other authors, carried out on *S. coeruleoalba* [12,29,30,31] and *D. delphis* [10,32] from different areas of the Mediterranean Sea (Table 5).

## 4. Discussion

The presence of metals and metalloids in all *S. coeruleoalba* and *D. delphis* samples, and their specific accumulation in the organs and tissues analyzed, represent useful data for evaluation of the health status and risk assessment for both dolphin species.

All toxic and essential metals were present in the highest levels in liver, due to its metabolic and detoxifying activity [33], except Cd, which was most abundant in kidney due to its specific tissue affinity. Intermediate values of metals were found in muscle. Pb showed, instead, a similar trend in all different organs/tissues analyzed. 

Skin and lung, which are generally not much used as an animal metric in metals determination, instead, demonstrated to be very interesting for evaluation of metals pollution. In fact, the metals concentrations found in skin and in lung could be correlated with acute exposure due to marine environmental pollution, considering also their important role as a secondary route of elimination for xenobiotics; instead, metals concentrations in liver, muscle and kidney are considered indicative of chronic exposure due to feeding [34]. Dolphins are able to bioaccumulate high levels of contaminants through the diet and biomagnify them according to the trophic transfer factor of the aquatic food chain, feeding on cephalopods, pelagic and demersal fishes [20,30], species considered accumulators of toxic metals present in sediment, sea bottom and superficial waters [7]. The role of feeding in metals accumulation could explain the high levels of Hg found, because this metal can reach total concentrations (inorganic and organic) in marine mammalians from 10 to 100 times higher than predatory fishes [2,6,14,15], due to their reduced detoxifying and excreting activity of xenobiotics [35].

The comparative analysis of toxic metals and metalloids (Hg, Pb, Cd, and As) carried out according to sex showed that the highest levels were present generally in females. These differences between the sexes, observed also by other authors [36], could be due to specific biotic factors: nutritional and health status [37], feeding habits [38], size (generally greater in females), and physiological factors [11,39]. In females, there is a higher amount of adipose tissue (which is a site of accumulation and reserve of xenobiotics) [40] and different hormonal activities, such as high levels of endogenous steroids (able to influence the kinetics of xenobiotics), etc. [41,42,43]. In males, by contrast, there is a rapid activation of enzymes of mixed oxidase function, responsible for rapid excretion of xenobiotics [44].

The comparison relating to the developmental stage of dolphins showed highest levels of toxic metals and metalloids in adults of both species. Several age-related factors can be responsible for metals accumulation in adults during their long life, particularly metabolic changes such as reduced activity of metabolizing enzymes, pathologic conditions of the liver, etc., with possible bioaccumulation in organs and tissues and relative toxicity [45].

Instead, Pb was most abundant in juveniles, probably due to maternal transfer during gestation and/or lactation [46,47,48]: in fact, as a consequence of hormonal changes in females, xenobiotics may be mobilized from deposition sites and released in the bloodstream [49], reaching the blood-placental and mammary barrier.

Relating to Hg, which was present in higher concentrations than the other metals and metalloids particularly in liver and skin, a risk assessment was carried out on dolphin health using the liver toxicity threshold, corresponding to 60 µg/g w.w. for mammalian species, and used as expression of liver damage [50]. However, Hg levels found in the liver of *S. coeruleoalba* (50.45 ± 7.24 µg g^−1^) and *D. delphis* (26.85 ± 5.32 µg g^−1^) corresponded to a low percentage of threshold toxicity in both species (43.66% and 30.85%, respectively).

For further toxicological evaluation, Hg levels were correlated with those of essential metals (Se and Zn), expressed as molar ratios (Table 4), to assess their protective role against mercury toxicity. The ^82^Se/^201^Hg molar ratios were lower than 1 (the value considered as a protection index) in all organs/tissues analyzed, except kidney, in accordance with other studies carried out on *S. coeruleoalba* [30] and *D. delphis* [11] from different areas of the Mediterranean Sea. Similarly, the molar ratio of ^66^Zn/^201^Hg, useful for evaluating a possible synergic effect with that of Se, was <1 and in particular lower in liver and skin of both species; instead, in muscle and kidney of *D. delphis* it was close to and greater than 1, respectively. In general, molar ratios lower than 1 indicate that an essential element such as Se, present in detoxification enzymatic systems, is insufficient to exercise a protective action against Hg toxicity. This molar ratio is commonly used by several authors in teleosts, sharks, and marine mammalians, to assess the risk of Hg exposure in both marine species and consumers of fishes [51,52,53], considering also the specific organ/tissue affinity of metals [54]. Although Hg shows a great affinity to bind Se, this ratio does not determine Se effectiveness [55] because several mechanisms could affect the Se-Hg interactions, such as Se sequestration, biochemical interactions, Se nutritional deficiencies [56,57], inhibition of selenium enzymes, etc. However, considering the presence of several essential metals (Se, Zn, etc.) in mammalians with antioxidant effects and competition for binding sites, they can exercise a synergic effect against Hg toxicity.

The exposure of dolphins to high Hg levels, however, is directly correlated to its presence as a natural component of the Mediterranean Sea bottom, but also to the pollution of this semi-enclosed basin, which due to its particular structure promotes accumulation and concentration of heavy metals and other pollutants [4]. However, the values of MPI, a parameter used to assess the metals pollution present in the aquatic environment and their transfer inside organs and tissues of various marine species (clams and also predatory fishes and marine mammalians) [4,58,59], showed values <1 (safety level) in all tissues analyzed (Figure 5), suggesting low contamination of the Mediterranean Sea where these dolphins lived.

Finally, from the comparative analysis of results of the present study with those of other authors (all expressed as mean values µg g^−1^ w.w. ± S.D.) in various organs/tissues of *S. coeruleoalba* [11,12,29,30] and *D. delphis* [9,31] from several areas of the Mediterranean Sea (Table 5), a similar trend of metals content was observed, except for Hg with intermediate values in *S. coeruleoalba*. Soma authors [11] also found Hg levels lower than other authors [36], hypothesizing that these data were due to a reduction of Hg in the ecosystem [50], although the most important factors able to influence metals content in the organs of marine mammalians are habitat (depth and distance from the coasts) [29], feeding habits, age and sex [38], etc. Therefore, considering our data and those in the recent literature, the changes observed in the trend of toxic metals and metalloids (particularly Hg) in dolphins of the Mediterranean Sea could be correlated to the reduced marine environmental pollution in recent decades, regulated by new European Directives on Hg and emissions of contaminants [60,61].

## 5. Conclusions

*S. coeruleoalba* and *D. delphis* are abundant marine species in the Mediterranean Sea and the trace metals present in their organs/tissues, particularly Hg, could represent a possible risk for health, though they have declined in recent times.

Our data on the metals and metalloids content in both dolphin species, according to sex and state of development, show the highest levels generally in females vs. males and in adults vs. juveniles, and significant differences relating to morphometric parameters and the organs/tissues analyzed.

Although metals exposure in dolphins, as reported in this study, is mainly correlated to marine environment, MPI values were <1 (safety level) in all organs/tissues analyzed, indicating a low contamination of the Mediterranean basin.

Furthermore, considering the exposure to high Hg levels in their habitat and the absorption of both toxic and essential metals, also during feeding, the molar ratios ^82^Se/^201^Hg and ^66^Zn/^201^Hg were analyzed. These ratios, lower than 1 (protective value) in most organs/tissues, indicate that Se and Zn are unable to exercise a sufficient protective role to counteract Hg toxicity. However, further studies are needed to better explain the real contribution of essential metals in Hg detoxification for the health of dolphins.

In conclusion, the study of metals and metalloids content in *S. coeruleoalba* and *D. delphis*, considered sentinel species of the Mediterranean Sea, is very important to verify the pollution trend of the aquatic environment in which they live, and, at the same time, the health of these marine species.

## Figures and Tables

**Table 1:** Sampling of *Stenella coeruleoalba* (n = 13) and *Delphinus delphis* (n = 12), beached along the Sicilian coastline of the Mediterranean Sea, collected by Istituto Zooprofilattico Sperimentale della Sicilia “A. Mirri”, Palermo (Italy).

Samples	Collection Site	N.	Sex	^a^ State of Conservation of Beached Carcasses	Weight (kg)	Length (m)	Development Stage (Age)
*Stenella* *coeruleoalba*	Palermo	2	1F	2	Range: 35.5–69	Range: 1.35–1.89	1 Juvenile
			1M	3			1 Adult
	Siracusa	6	3F	2	Range: 14.5–80	Range: 0.98–2.08	3 Juveniles
			3M	2			3 Adults
	Messina	5	3F	2	Range: 20.7–61	Range: 1.02–1.95	2 Juveniles
			2M	3			3 Adults
*Delphinus* *delphis*	Palermo	12	7F	2	Range: 14.0–81.5	Range: 1.11–2.05	5 Juveniles
			5M	2			7 Adults

^a^ State of conservation of beached carcasses, according to scoring system: 1—alive/just deceased, 2—fresh carcass, 3—moderately decomposed carcass, 4—advanced decomposed carcass, 5—mummified carcass or skeletal remains.

**Table 2:** Validation parameters of analytical method in ICP-MS.

Metal Elements	Linearity (R^2^)	LOD ^a^ (ng/g)	LOQ ^b^ (ng/g)	Certified Values ^c^ (µg g^−1^ w.w.)	Measured Values (µg g^−1^ w.w.)	Recovery (%)	RSD ^d^ (%)
Hg	0.99	0.02	0.04	0.44 ± 0.18	0.33 ± 0.08	(99.89)	1.12
Pb	1.00	0.14	1.39	0.16 ± 0.03	0.15 ± 0.02	(98.97)	0.42
Cd	0.99	0.02	0.03	14.52 ± 0.6	14.11 ± 0.43	(99.99)	1.77
As	0.99	0.01	0.02	34.66 ± 2.4	33.84 ± 0.32	(99.99)	1.27
Se	0.999	0.02	0.06	8.31 ± 1.82	7.75 ± 0.68	(98.89)	1.45
Zn	0.99	0.01	0.07	105.31 ± 5.04	108.22 ± 8.04	(99.95)	4.05

^a^ LOD: limit of detection; ^b^ LOQ: limit of quantification; ^c^ Certified Reference Material of Dogfish Liver for Trace Metals (DOLT-5); ^d^ RSD (%): relative standard deviation, averaging four individual determinations.

**Table 3:** Toxic and essential metals and metalloids, expressed as mean value ± S.D. µg g^−1^ (w.w.) of individual measurements, in organs/tissues of *Stenella coeruleoalba* (n = 13) and *Delphinus delphis* (n = 12) from Sicilian coastlines of the Mediterranean Sea.

Elements	Samples	Skin	Liver	Muscle	Lung	Kidney
		(M.V. ± S.D.)	(M.V. ± S.D.)	(M.V. ± S.D.)	(M.V. ± S.D.)	(M.V. ± S.D.)
Toxic						
**Hg**	*Stenella coeruleoalba*	50.45 ± 7.24 **^#^	26.20 ± 7.12 *^#^	9.11 ± 3.54	8.97 ± 1.35	*-*
	*Delphinusdelphis*	26.85 ± 5.32 ^#^	18.51 ± 5.82 ^#^	7.07 ± 1.53	*-*	0.96 ± 0.38
**Pb**	*Stenella coeruleoalba*	0.14 ± 0.05	0.16 ± 0.07	0.15 ± 0.11 *	0.18 ± 0.09	*-*
	*Delphinus delphis*	0.14 ± 0.03	0.15 ± 0.05	0.51 ± 0.05	*-*	0.03 ± 0.01
**Cd**	*Stenella coeruleoalba*	0.07 ± 0.01 *	0.42 ± 0.07 ^#^	0.06 ± 0.03	0.03 ± 0.01	*-*
	*Delphinus delphis*	0.04 ± 0.02	0.21 ± 0.06 *^#^	0.03 ± 0.01 *	*-*	1.20 ± 0.22
**As**	*Stenella coeruleoalba*	0.22 ± 0.03	0.28 ± 0.06 *	0.07 ± 0.01	0.09 ± 0.08	*-*
	*Delphinus delphis*	0.36 ± 0.04	0.16 ± 0.05	0.18 ± 0.02 *	*-*	nd
Essential						
**Se**	*Stenella coeruleoalba*	4.65 ± 0.08	8.59 ± 0.04	5.29 ± 0.02	3.22 ± 0.03	*-*
	*Delphinus delphis*	3.52 ± 0.11	6.58 ± 0.24	2.06 ± 0.06	*-*	1.08 ± 0.32
**Zn**	*Stenella coeruleoalba*	10.67 ± 1.65	11.59 ± 2.03	6.91 ± 0.56	5.14 ± 0.21	*-*
	*Delphinus delphis*	7.64 ± 1.58	9.61 ± 1.55	5.85 ± 0.38	*-*	2.17 ± 0.41

* *p <* 0.05, ** *p <* 0.001 vs. other species; **^#^** *p <* 0.001 vs. other organs/tissues; - = missing sample; nd = not detectable value.

**Table 4:** Molar ratio (a) ^82^Se/^202^Hg and (b) ^66^Zn /^202^Hg in organ/tissues of *Stenella coeruleoalba* and *Delphinus delphis* from the Mediterranean Sea.

Organ/Tissue	Ratio	*Stenella coeruleoalba*	*Delphinus* *delphis*
Lung	^82^Se/^201^Hg	0.35	-
	^66^Zn/^201^Hg	0.57	-
Muscle	^82^Se/^201^Hg	0.58	0.29
	^66^Zn/^201^Hg	0.75	0.82
Liver	^82^Se/^201^Hg	0.32	0.35
	^66^Zn/^201^Hg	0.44	0.51
Skin	^82^Se/^201^Hg	0.09	0.13
	^66^Zn/^201^Hg	0.21	0.28
Kidney	^82^Se/^201^Hg	-	1.12
	^66^Zn/^201^Hg	-	2.26

**Table 5:** Comparison of metals and metalloids levels (mean values mg/kg w.w. ± S.D.) in tissues of *S. coeruleoalba* and *D. delphis* from our study with those present in the literature from different areas of the Mediterranean Sea.

Dolphin Species	Location	Tissues	Hg	Cd	As	Se	Zn	References
*Stenella coeruleoalba*	Sicilian coasts of the Mediterranean Sea	Liver	26.20 ± 7.12	0.42 ± 0.07	0.28 ± 0.06	8.59 ± 0.02	11.59 ± 2.03	Our data
		Muscle	9.11 ± 3.55	0.06 ± 0.03	0.07 ± 0.01	5.29 ± 0.02	6.91 ± 0.05	
		Skin	50.45 ± 7.24	0.07 ± 0.01	0.02 ± 0.01	4.65 ± 0.02	10.67 ± 0.06	
		Lung	8.97 ± 1.35	0.03 ± 0.01	0.09 ± 0.04	3.22 ± 0.01	5.14 ± 0.02	
	Ligurian Sea (Italy)	Liver	83 ± 41	0.94 ± 0.53	-	39 ± 27	240 ± 30.7	[29]
		Muscle	7.3 ± 7.3	0.05 ± 0.03	-	2.0 ± 2.1	191 ± 43	
		Kidney	7.8 ± 8.4	5.2 ± 2.7	-	5.2 ± 1.0	124 ± 63	
	Israeli Mediterranean coasts	Liver	134 ± 89	6.4 ± 4.24	0.75 ± 2.9	47 ± 28	69 ± 43	[30]
		Muscle	8.5 ± 10.4	0.11 ± 0.15	0.19 ± 0.52	3.2 ± 3.75	23.2 ± 17.6	
		Kidney	11.4 ± 6.4	14 ± 8	0.4 ± 0.3	6.5 ± 2.6	30.9 ± 8.7	
	Galician coasts of Spain (North Atlantic)	Liver	22.9 ± 39.1	3.4 ± 3.8	<0.67	12.3 ± 17.2	53 ± 21.1	[12]
		Kidney	2.8 ± 2.6	12.3 ± 11	<0.67	3.2 ± 1.4	24.2 ± 7.5	
	Murcia region (Spain)	Liver	139.53 ± 62.23	0.84 ± 0.68	1.23 ± 1.38	50.53 ± 60.05	-	[11]
		Muscle	8.14 ± 9.50	n.d ± 0.01	1.05 ± 5.37	3.98 ± 3.81	-	
		Kidney	9.93 ± 7.67	3.16 ± 1.93	0.76 ± 1.44	6.82 ± 3.51	-	
		Lung	7.80 ± 8.52	0.04 ± 0.04	0.23 ± 0.28	6.58 ± 3.61	-	
*Delphinus delphis*	Sicilian coasts of the Mediterranean Sea	Liver	18.51 ± 5.32	0.21 ± 0.06	0.16 ± 0.05	6.58 ± 0.04	9.61 ± 1.55	Our data
		Muscle	7.07 ± 1.53	0.03 ± 0.01	0.18 ± 0.02	2.06 ± 0.02	5.85 ± 0.38	
		Skin	26.85 ± 9.13	0.04 ± 0.02	0.36 ± 0.04	3.52 ± 0.11	7.64 ± 1.58	
		Kidney	0.96 ± 3.35	1.20 ± 0.22	-	1.08 ± 0.32	2.17 ± 0.41	
	Western Portuguese coasts (2009–2013)	Liver	16.7 ± 2.9	0.4 ± 0.06	0.6 ± 0.2	7.4 ± 1.01	46.7 ± 25	[10]
		Muscle	2.1 ± 0.2	2.3 ± 0.3	0.4 ± 0.03	4.0 ± 0.2	21.4 ± 0.7	
		Kidney	0.9 ± 0.08	± 0.00	0.3 ± 0.02	0.8 ± 0.05	10.9 ± 0.4	
	Portuguese coasts	Liver	11 ± 18.3	-	58.8 ± 21.6			[31]
		Muscle	0.80 ± 0.70	-	23.4 ± 4.56			
		Kidney	1.63 ± 1.44	0.06 ± 0.55	16.80 ± 8.54			

## Data Availability

All data and results related to this study are original and included in the article.
